# Elamipretide Attenuates Pyroptosis and Perioperative Neurocognitive Disorders in Aged Mice

**DOI:** 10.3389/fncel.2020.00251

**Published:** 2020-08-13

**Authors:** Youmei Zuo, Lei Yin, Xinqi Cheng, Jun Li, Hao Wu, Xuesheng Liu, Erwei Gu, Jing Wu

**Affiliations:** ^1^Department of Anesthesiology, First Affiliated Hospital of Anhui Medical University, Hefei, China; ^2^Key Laboratory of Anesthesiology and Perioperative Medicine of Anhui Higher Education Institutes, Anhui Medical University, Hefei, China; ^3^Department of Anesthesiology, Subei People’s Hospital of Jiangsu Province, Yangzhou, China; ^4^Jiangsu Key Laboratory of Molecular Medicine, Medical School of Nanjing University, Nanjing, China

**Keywords:** perioperative neurocognitive disorders, elamipretide, mitochondria, pyroptosis, neuroinflammation

## Abstract

Pyroptosis is a recently characterized inflammatory form of programmed cell death that is thought to be involved in the pathogenesis of perioperative neurocognitive disorders (PND). Elamipretide (SS-31), a mitochondrial-targeted peptide with multiple pharmacological properties, including anti-inflammatory activity, has been demonstrated to protect against many neurological diseases. However, the effect of elamipretide on pyroptosis in PND has not been studied. We established an animal model of PND by performing an exploratory laparotomy on mice under isoflurane anesthesia and examined the effects of elamipretide on cognitive function, synaptic integrity, neuroinflammation, mitochondrial function, and signaling controlling pyroptosis. Our results showed that anesthesia and surgery caused mitochondrial dysfunction and abnormal morphology, activation of canonicalnod-like receptor pyrin domain-containing 3 (NLRP3) inflammasome-caspase-1 dependent pyroptosis, and downregulation of synaptic integrity-related proteins in the hippocampus in aged mice, thus leading to learning and memory deficits in behavioral tests. Remarkably, treatment with the mitochondrial-targeted peptide elamipretide not only had protective effects against mitochondrial dysfunction but also attenuated surgery-induced pyroptosis and cognitive deficits. Our results provide a promising strategy for the treatment of PND involving mitochondrial dysfunction and pyroptosis.

## Introduction

Perioperative neurocognitive disorders (PND), encompassing acute delirium and longer-lasting postoperative cognitive dysfunction, are the most common and least recognized complications of surgery and anesthesia in older patients (Subramaniyan and Terrando, 2019; Eckenhoff et al., 2020). The incidence of PND varies from 41 to 75% at 7 days to 18–45% at 3 months post-surgery (Deo et al., 2011; Skvarc et al., 2018). Patients with PND are at increased risk of mortality and morbidity, reduced quality of life, and substantial healthcare costs (Inouye et al., 2014; Subramaniyan and Terrando, 2019). However, the pathophysiology of PND remains largely unknown.

Increasing evidence suggests that neuroinflammation is a key contributor in PND (Saxena et al., 2019; Subramaniyan and Terrando, 2019). Pyroptosis is a recently characterized inflammatory form of programmed cell death. This type of cell death can be triggered through the canonical nod-like receptor pyrin domain-containing 3 (NLRP3) inflammasome-caspase-1 pathway and non-canonical caspase-4/5/11 pathway. More specifically, active caspase-1 or caspase-4/5/11 enzymatically cleaves Gasdermin D (GSDMD) protein, the executor of pyroptosis, into two fragments (the N domain and C domain). The GSDMD N domain then forms pores in lipid membranes and induces pyroptosis through cell membrane disruption (Shi et al., 2017; Kesavardhana et al., 2020). Pyroptosis has been implicated in the pathogenesis of many inflammatory and non-inflammatory diseases (Man et al., 2017; Voet et al., 2019). However, little is known about its role in PND.

NLRP3 inflammasome activation leads to caspase-1-dependent pyroptosis and secretion of proinflammatory cytokines, such as interleukin-1β (IL-1β) and IL-18. Mitochondria are important organelles in cells that function in not only energy production but also diverse metabolic pathways. Recent studies have identified new roles of mitochondria in the initiation and regulation of the NLRP3 inflammasome (Alfonso-Loeches et al., 2014; Yu and Lee, 2016; Liu et al., 2018; Wei et al., 2019). Mitochondria serve as a platform for inflammasome assembly. Also, dysfunctional mitochondria release several stimulating factors that activate the NLRP3 inflammasome. Notably, mitochondrial dysfunction has been associated with the earliest stages in PND pathogenesis (Wu et al., 2015a; Chen et al., 2018; Zhao et al., 2019). Therefore, agents that protect mitochondria may have great promise in the treatment of PND by inhibiting activation of the NLRP3 inflammasome and pyroptosis.

Elamipretide (SS-31, D-Arg-dimethylTyr-Lys-Phe-NH_2_) is a novel mitochondria-targeted peptide. Its alternating aromatic-cationic structure allows it to freely cross the blood-brain barrier and cell membranes; consequently, it can concentrate >1,000-fold in the mitochondrial inner membrane independently of the mitochondrial membrane potential (MMP; Szeto, 2006, 2014). Previous studies have shown that elamipretide protects mitochondrial function by decreasing the generation of reactive oxygen species (ROS) and maintaining adenosine triphosphate (ATP) production and MMP levels in many animal models (Wu et al., 2015a,b; Zhao et al., 2019). Elamipretide has also been reported to reduce defects in mitochondrial dynamics and to enhance mitochondrial biogenesis in neurons in Huntington’s disease (Yin et al., 2016). However, the effects of elamipretide on mitochondria-mediated activation of the NLRP3 inflammasome and pyroptosis have not been reported.

In the present study, we used a mouse model involving exploratory laparotomy under isoflurane anesthesia to mimic clinical human abdominal surgery. This preclinical model has been shown to induce neuroinflammation and cognitive deficits and has been widely used to investigate the molecular basis of PND and to test potential therapeutic strategies (Qiu et al., 2020; Wen et al., 2020). We hypothesized that: (1) anesthesia and surgery would cause mitochondrial dysfunction and trigger the activation of NLRP3 inflammasome-caspase-1 dependent pyroptosis in the hippocampus in aged mice, thus contributing to PND pathogenesis; and (2) administration of elamipretide would protect against surgery-induced cognitive deficits by attenuating mitochondrial dysfunction and neuronal pyroptosis.

## Materials and Methods

### Animals

C57BL/6 male mice at 15 months of age were purchased from the Model Animal Research Center of Nanjing University, Nanjing, China. The experiments were started after mice had acclimated to the environment for 7 days. Mice were housed in groups of five individuals per cage under controlled conditions (23–25°C and 50 ± 10% humidity, 12:12 h light:dark cycle) and were given free access to food and water. All experimental procedures in this study were approved by the local ethics committee for animal research at Anhui Medical University and were performed according to the Guidelines for the Care and Use of Laboratory Animals from the National Institutes of Health, Bethesda, MD, USA.

### Experimental Protocols

The mice were randomly assigned to one of the following four treatment protocols: control + vehicle (Veh group), control + elamipretide (Ela group), surgery + vehicle (Sur group), and surgery + elamipretide (Sur+Ela group). Elamipretide (5 mg/kg, synthesized in China by Peptides Company Limited, Shanghai) or normal saline (vehicle) was intraperitoneally administered to mice 30 min before isoflurane anesthesia and once daily for three consecutive days thereafter. The dose of elamipretide was selected according to previous research (Wu et al., 2015a,b; Zhao et al., 2019).

Exploratory laparotomy was performed under aseptic conditions with isoflurane anesthesia, as described in previous studies (Qiu et al., 2020; Wen et al., 2020). This model has been used in studies investigating both postoperative delirium (Peng et al., 2016; Lu et al., 2020) and postoperative cognitive dysfunction (Kawano et al., 2015; Qiu et al., 2020), and is referred to as a model for PND in this study, according to the nomenclature recently adopted in major anesthesiology journals. Briefly, mice were anesthetized with 1.8% isoflurane and oxygen at 2 L/min in an induction chamber for 30 min. After the abdominal region was shaved and cleaned, a 1 cm median abdominal incision was made, and the peritoneal cavity was penetrated. Then, the viscera, intestines, and musculature were explored, and approximately 10 cm of the small intestine was exteriorized from the peritoneal cavity, covered with moist gauze and manipulated manually. Finally, the peritoneal cavity was sutured layer by layer with sterile 4–0 chromic gut sutures. The surgical procedure was performed in mice anesthetized with 1.5% isoflurane and sustained for 20 min. For the control mice, neither anesthesia nor surgery was performed.

### Tissue Collection

Six mice in each group were decapitated on postoperative day 4, and the brain was rapidly removed and separated into two halves for biochemical assays and histochemical analysis. One half of the hippocampal tissue was quickly dissected on ice and cut into pieces for mitochondria isolation and western blotting and enzyme-linked immunosorbent assay (ELISA) analysis. For histochemical experiments, the other half of each brain was fixed in 4% paraformaldehyde overnight at 4°C and embedded in paraffin. The tissues were sliced into 4 mm sections before use.

### Mitochondrial Isolation

The hippocampal tissues were homogenized in ice-cold Dounce homogenizers (1:10, w/v) with isolation buffer (Beyotime Institute of Biotechnology, Shanghai, China) and centrifuged at 1,000× *g* for 5 min at 4°C. Supernatants were removed and centrifuged at 12,000× *g* for 10 min at 4°C to obtain pure cytosolic fractions. The mitochondria-enriched pellets were gently resuspended and re-pelleted by centrifugation at 12,000× *g* for 10 min. The concentrations of mitochondrial protein and cytosolic protein were determined with a Micro BCA protein assay kit (Beyotime Institute of Biotechnology) with bovine serum albumin as the standard.

### Determination of ATP, MMP, and ROS Levels

ATP content was measured with a firefly luciferase-based ATP assay kit (Beyotime Institute of Biotechnology, Shanghai, China). MMP levels were determined with a 5,5′,6,6′-tetrachloro-1,1′,3,3′ tetraethylbenzimidazolylcarbocyanine iodide (JC-1) MMP detection kit (Beyotime Institute of Biotechnology, Shanghai, China), and intracellular ROS were detected with a ROS assay kit (Genmed Scientifics Inc., Shanghai, China) containing an oxidation-sensitive fluorescent probe (DCFH-DA). All measurements were performed according to the manufacturer’s instructions. Data were normalized to the control group values and expressed as a percentage of control levels.

### Western Blotting Analysis

Proteins (40 μg per lane) were separated with SDS-PAGE and then transferred to a polyvinylidene fluoride membrane (Millipore, Billerica, MA, USA). After being blocked with 5% skim milk in TBST for 1 h, the membranes were incubated with primary antibody against Dynamin-related protein 1 (DRP1; 1:1,000; Abcam), Mitofusin 2 (MFN2; 1:1,000; Abcam), NLRP3 (1:1,000; Cell Signaling Technology, Danvers, MA, USA), caspase-1 (1:1,000; Santa Cruz Biotechnology, Santa Cruz, CA, USA), GSDMD (1:500; Abcam), interleukin-1β (IL-1β; 1:200; Santa Cruz Biotechnology, Santa Cruz, CA, USA), IL-18 (1:200; Santa Cruz Biotechnology, Santa Cruz, CA, USA), synapsin 1 (1:2,500; Millipore, Billerica, MA, USA), PSD-95 (1:1,500; Abcam), voltage-dependent anion channel (VDAC; 1:1,000; Cell Signaling Technology, Danvers, MA, USA), or GAPDH (1:1,000; Cell Signaling Technology, Danvers, MA, USA). After three washes, the membranes were treated with species-specific peroxidase-conjugated secondary antibodies for 1 h at room temperature. Bands were visualized by enhanced chemiluminescence and quantified with Image Quant Software (Syngene).

### Enzyme-Linked Immunosorbent Assay

The quantification of IL-1β and IL-18 in the hippocampus was performed with an ELISA kit according to the manufacturer’s instructions (R&D Systems, Inc., Minneapolis, MN, USA). Results are reported as pictograms per milliliter total protein.

### Immunohistochemistry (IHC)

Immunohistochemistry (IHC) was used to detect the immunoreactivity of caspase-1. Serial slides were rinsed in PBS and blocked in 5% BSA for 1 h, then reacted with an antibody against caspase-1 (1:100; Santa Cruz Biotechnology, Santa Cruz, CA, USA) for 2 h. The sections were then incubated with a secondary antibody labeled with horseradish peroxidase for 30 min at room temperature. Cells with brownish-yellow cytoplasm were counted as positive cells. For quantitative immunostaining, caspase-1-positive cells were observed under an inverted microscope, and the CA1 region was counted for all groups in ImageJ software.

### Hematoxylin and Eosin (HE) Staining and TUNEL Assays

Hematoxylin and Eosin (HE) staining and terminal deoxynucleotidyl transferase-mediated dUTP nick end labeling (TUNEL) assays were used to examine neuronal damage. Hippocampal samples were stored in a 4% paraformaldehyde solution and embedded in paraffin. Then, the paraffin sections (4 μm) were dewaxed and rehydrated in differential alcohol gradients for subsequent HE staining. The histopathological damage in the CA1 region of the hippocampus was determined under standard light microscopy and evaluated on a standard semi-quantitative scale (Tang et al., 2016; Fan et al., 2018).

To detect the degree of DNA fragmentation, we performed TUNEL assays with a commercial kit (Roche TUNEL kit, Roche Molecular Biochemicals, Germany) following the manufacturer’s instructions. The number of TUNEL-positive cells in the CA1 region of the hippocampus was observed under an inverted microscope. TUNEL-positive cells appeared dark brown.

### Electron Microscopy

Ultrastructural changes in the hippocampal mitochondria were assessed with transmission electron microscopy (Wu et al., 2017). Slices were stained with 4% uranyl acetate—0.3% lead citrate and observed with a Tecnai G2 Transmission Electron Microscope (FEI Company, Hillsboro, OR, USA). Electron micrographs were analyzed in ImageJ software. Four neurons per animal were selected for electron microscopy analysis. Morphometric analyses were conducted by an investigator blinded to the experimental conditions.

### Open-Field Tests

To evaluate anxiety behavior and general locomotor activity, we subjected mice (*n* = 12 for each group) to open field tests on the postoperative day 7. Each mouse was gently placed at the center of a black plastic chamber (50 cm × 50 cm × 50 cm) for 5 min, and exploratory behavior was automatically recorded by a video tracking system (XR-XZ301, Shanghai Soft Maze Information Technology Company Limited, Shanghai, China). The total distance traveled and the time spent in the center area of the chamber was measured. After each test, the arena was cleaned with 75% alcohol to avoid the presence of olfactory cues.

### Fear Conditioning Tests

To investigate learning and memory function, we subjected mice to fear conditioning tests (XR-XC404; Shanghai Softmaze Information Technology Company Limited, Shanghai, China) 2 h after the open field tests. Each mouse was placed into a conditioning chamber and allowed to explore freely for 3 min. Then one tone-foot-shock pairing (tone, 30 s, 70 dB, 3 kHz; foot-shock, 3 s, 0.7 mA) was delivered. The mouse then stayed in the chamber for another 30 s and was then returned to the home cage. The contextual fear conditioning test (a hippocampus-dependent task) was performed 24 h later by placing each mouse back in the same test chamber for 5 min without any stimulation. Two hours later, the tone fear conditioning test (a hippocampus-independent task) was performed by placing each mouse in a novel chamber with a different shape, color, and smell from the previous chamber, and the same tone was presented for 3 min without foot shock. Freezing behavior, defined as the absence of all visible movement except for respiration, was automatically recorded by the video tracking system.

### Statistical Analysis

Data are presented as mean ± SEM and were analyzed in the Statistical Product for Social Sciences (SPSS; version 17.0, IL, USA) software. The differences between groups were determined by one-way analysis of variance followed by Tukey’s test. A *p*-value <0.05 was considered to indicate statistical significance.

## Results

### Elamipretide Protects Against Surgery-Induced Mitochondrial Dysfunction and Abnormal Morphology in the Hippocampus in Aged Mice

**Figure 1:**
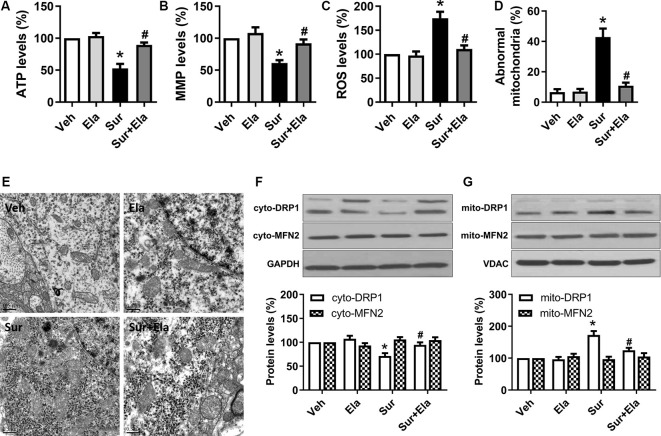
Protective effects of elamipretide on mitochondrial function and morphology in the hippocampus in aged mice after surgery. Elamipretide (5 mg/kg) or normal saline (vehicle) was intraperitoneally administered to mice 30 min before isoflurane anesthesia and once daily for three consecutive days thereafter. The adenosine triphosphate (ATP) production **(A)**, mitochondrial membrane potential (MMP) levels **(B)**, and reactive oxygen species (ROS) generation **(C)** were determined with fresh hippocampal tissue homogenates obtained on postoperative day 4. **(D)** Quantification of abnormal mitochondria in hippocampal neurons of the aged mice in each group. **(E)** Representative images of mitochondrial ultrastructure in hippocampal neurons of aged mice on postoperative day 4. Scale bar = 0.5 μm for all photographs. **(F,G)** Representative western blotting and quantitative analysis of protein levels of Dynamin-related protein 1 (DRP1) and Mitofusin 2 (MFN2) in the isolated cytosolic (cyto-) and mitochondrial (mito-) fractions of hippocampal tissue homogenates. Values are presented as mean ± SEM (*n* = 6 mice/group). *p < 0.05 vs. the Veh group; ^#^*p* < 0.05 vs. the Sur group.

Previous work has demonstrated that elamipretide 5 mg/kg i.p. administration improves mitochondrial function in the hippocampus in mice under isoflurane anesthesia or in lipopolysaccharide-treated mice (Wu et al., 2015a,b; Zhao et al., 2019). In line with these data, our study showed that elamipretide 5 mg/kg i.p. administration improved mitochondrial function by increasing ATP production (Figure 1A) and MMP levels (Figure 1B) and decreasing ROS generation (Figure 1C) in the hippocampus in aged mice after surgery. Moreover, elamipretide administration protected mitochondria against morphological damage after surgery. The electron microscopy results showed that the number of abnormal mitochondria (Figure 1D) was significantly greater in the surgery group, many of the abnormal mitochondria had disorganized cristae and disrupted outer membranes, and other mitochondria were dark and condensed (Figure 1E). In contrast, mitochondria in the elamipretide-treated surgery group appeared similar to those in the control group without ultrastructural damage (Figure 1E).

Mitochondria are dynamic organelles that undergo continuous fission and fusion. DRP1 and MFN2 are two key regulators that maintain these processes (Scott and Youle, 2010; Westrate et al., 2014). After activation, DRP1 is translocated from the cytosol to the mitochondrial outer membrane, where it mediates mitochondrial fission. MFN2 regulates mitochondrial outer membrane fusion. Accordingly, we measured the expression of DRP1 and MFN2 in both the cytosolic and mitochondrial compartments. The western blotting results showed that surgery-induced a decrease in DRP1 expression in the cytosolic fraction (Figure 1F) and on increase in DRP1 expression in the mitochondrial fraction (Figure 1G), as compared with those under the control condition at postoperative day 4, thus suggesting that DRP1 translocated into the mitochondria, and excessive mitochondrial fission occurred. However, surgery did not significantly alter the expression of MFN2 in either the cytosolic fraction of the mitochondrial fraction. Notably, the administration of elamipretide reversed the transport of DRP1 from the cytosol to the mitochondria and inhibited surgery-induced mitochondrial fission.

Collectively, our results suggested that elamipretide protected against surgery-induced mitochondrial dysfunction and abnormal morphology in the hippocampus in aged mice.

### Elamipretide Inhibits Surgery-Induced Activation of the NLRP3 Inflammasome-Caspase-1 Pathway in the Mouse Hippocampus

**Figure 2:**
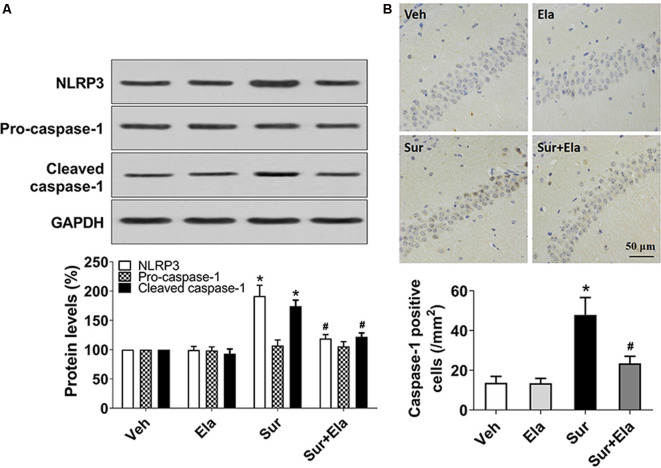
Effects of elamipretide on the canonical nod-like receptor pyrin domain-containing 3 (NLRP3) inflammasome-caspase-1 pathway in the hippocampus in aged mice after surgery. **(A)** Representative western blotting and quantitative analysis of protein levels of NLRP3, Pro-caspase-1, and cleaved caspase-1 in the hippocampus in aged mice. **(B)** Representative images of caspase-1 Immunohistochemistry (IHC) staining in the hippocampal CA1 region. Cells with brownish-yellow cytoplasm are positive for caspase-1. Scale bar = 50 μm for all photographs. The lower panel presents the number of caspase-1-positive neurons in the CA1 region of the hippocampus. Values are presented as mean ± SEM (*n* = 6 mice/group). *p < 0.05 vs. the Veh group; ^#^*p* < 0.05 vs. the Sur group.

Mitochondria have emerged as central regulators of NLRP3 inflammasome activation. The NLRP3 inflammasome is the best-characterized member of the NLRP family, which consists of the NLRP3 protein, apoptosis-associated speck-like proteins, and precursor caspase-1 (Pro-caspase-1). The NLRP3 inflammasome has major roles in PND through triggering the release of IL-1β and IL-18 *via* active caspase-1 (Fan et al., 2018; Fu et al., 2020). To investigate whether protecting mitochondria with elamipretide might inhibit surgery-induced activation of the NLRP3 inflammasome-caspase-1 pathway, we measured the protein levels of NLRP3, pro-caspase-1, and cleaved caspase-1, and the number of caspase-1 positive cells in the hippocampal CA1 area. Our results showed that administration of elamipretide reversed surgery-induced upregulation of NLRP3 and cleaved caspase-1 (Figure 2A), and decreased the number of caspase-1 positive cells in the CA1 area (Figure 2B), thus indicating that activation of the NLRP3 inflammasome-caspase-1 pathway was inhibited by elamipretide in the hippocampus in aged mice after surgery.

### Elamipretide Suppresses Surgery-Induced Pyroptosis and Neuroinflammation in the Mouse Hippocampus

**Figure 3:**
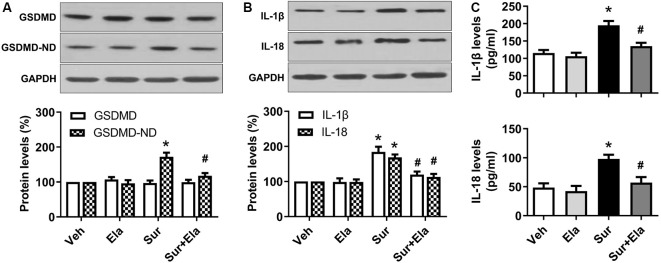
Effects of elamipretide on pyroptosis and neuroinflammation in the hippocampus in aged mice after surgery. **(A)** Representative western blotting and quantitative analysis of protein levels of the full-length Gasdermin D (GSDMD) and GSDMD-N domain (GSDMD-ND) in the hippocampus in aged mice. **(B)** Western blotting analyses of IL-1β and IL-18 levels. **(C)** Enzyme-linked immunosorbent assay (ELISA) analyses of IL-1β and IL-18 levels. Values are presented as mean ± SEM (*n* = 6 mice/group). *p < 0.05 vs. the Veh group; ^#^*p* < 0.05 vs. the Sur group.

GSDMD is the substrate of active caspase-1 and the executor of pyroptosis. The N domain of GSDMD can form pores in lipid membranes, thus resulting in cell membrane disruption and inflammatory cytokines release (Man et al., 2017; Shi et al., 2017). Our results showed that levels of the GSDMD N domain (Figure 3A) and inflammatory IL-1β and IL-18 (Figures 3B,C) in the hippocampus were markedly higher in the surgery group than the vehicle group, thus indicating that surgery-induced activation of NLRP3 inflammasome-caspase-1-dependent pyroptosis. Interestingly, the administration of elamipretide attenuated the activation of pyroptosis and the release of inflammatory cytokines (Figure 3). These results implied that elamipretide suppressed surgery-induced pyroptosis and neuroinflammation in the mouse hippocampus.

### Elamipretide Rescues Surgery-Induced Neuronal Damage and Synaptic Dysfunction in the Mouse Hippocampus

**Figure 4:**
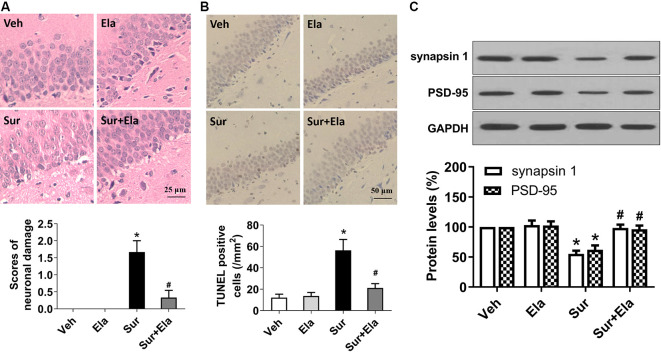
Elamipretide rescues surgery-induced neuronal damage and synaptic dysfunction in the mouse hippocampus. **(A)** Representative images of Hematoxylin and Eosin (HE) staining in the hippocampal CA1 region. Scale bar = 25 μm for all photographs. The lower panel shows statistical scores of neuronal damage. **(B)** Representative images of TUNEL staining in the hippocampal CA1 region. TUNEL-positive cells are dark brown. Scale bar = 50 μm for all photographs. The lower panel presents the number of TUNEL-positive neurons in the CA1 region of the hippocampus. **(C)** Representative western blotting and quantitative analysis of protein levels of synapsin 1 and PSD-95 in the hippocampus in aged mice. Values are presented as mean ± SEM (*n* = 6 mice/group). *p < 0.05 vs. the Veh group; ^#^*p* < 0.05 vs. the Sur group.

Pyroptosis is an important mechanism of neuronal cell death. The morphological characteristics of pyroptosis are cell swelling, positivity for Annexin V and TUNEL staining, chromatin condensation, and an absence of DNA laddering (Sharma and Kanneganti, 2016; Man et al., 2017; Fan et al., 2018). In the present study, HE staining revealed that mice in the surgery group had many neurons with fragmented cell bodies and extensive karyopyknosis and karyolysis (Figure 4A). Also, TUNEL assays showed that the number of TUNEL-positive neurons in the surgery group was greater than that in the vehicle group (Figure 4B). In contrast, elamipretide significantly attenuated surgery-induced neuronal damage in the hippocampal CA1 region (Figure 4).

To further determine the effects of elamipretide on surgery-induced synaptic dysfunction, we measured the protein levels of synapsin 1 and PSD-95, two important indicators of the structural and functional integrity of excitatory synapses (Mirza and Zahid, 2018). Our results showed that elamipretide attenuated the surgery-induced downregulation of synapsin 1 and PSD-95 (Figure 4C), thus suggesting a protective effect of elamipretide toward synaptic integrity. Collectively, our results showed that elamipretide rescued surgery-induced neuronal damage and synaptic dysfunction in the hippocampus in aged mice.

### Elamipretide Attenuates Surgery-Induced Cognitive Deficits in Aged Mice

**Figure 5:**
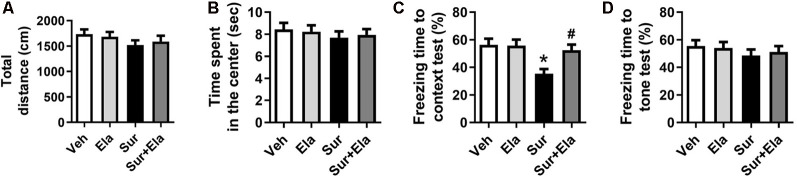
Elamipretide attenuates surgery-induced cognitive deficits in aged mice. **(A)** Total distance traveled and **(B)** time spent in the center in open field tests. The open field tests in aged mice were performed on postoperative day 7. **(C)** Freezing time to context and **(D)** freezing time to tone in the fear conditioning tests. Values are presented as mean ± SEM (*n* = 12 mice/group). *p < 0.05 vs. the Veh group; ^#^*p* < 0.05 vs. the Sur group.

Previous studies have shown that cognitive deficits induced by surgery in aged mice can be observed on postoperative day 7 (Fu et al., 2020; Qiu et al., 2020). We used open field tests to evaluate locomotor activity and exploratory behavior. There were no significant differences among the four groups in the open field tests, including the total distance (Figure 5A) and the time spent in the center (Figure 5B), thus suggesting that the surgery did not cause a spontaneous decline in locomotor activity. Then we evaluated learning and memory function *via* fear conditioning tests. In the contextual fear conditioning test, the percentage of freezing time was lower in the surgery group than in the vehicle group on the postoperative day 8 (Figure 5C), thus indicating that surgery evoked hippocampus-dependent cognitive deficits. There was no difference in the cued fear conditioning test results among the four groups (Figure 5D). Notably, the administration of elamipretide attenuated the surgery-induced cognitive deficits in aged mice by increasing the percentage of freezing time in the contextual fear conditioning test.

## Discussion

In the present study, we demonstrated that surgical laparotomy induced mitochondrial dysfunction and abnormal morphology, and activation of NLRP3 inflammasome-caspase-1 dependent pyroptosis, thereby leading to neuronal damage and impaired synaptic integrity in the hippocampus and contributing to cognitive deficits in aged mice. Of note, the mitochondria-targeted peptide elamipretide attenuated these abnormalities. Therefore, elamipretide may be a therapeutic strategy for treating neuroinflammation and PND.

Growing evidence implicates neuroinflammation in the pathophysiology of PND. Local inflammation due to surgical trauma is paralleled by an increase in systemic inflammatory mediators. Several of these mediators have been shown to influence inflammatory processes in the brain and to be followed by neuroinflammation and neuronal death, which can lead to cognitive deficits (Hovens et al., 2014; Schain and Kreisl, 2017; Saxena et al., 2019). Also, isoflurane anesthesia alone has been found to induce neuroinflammation and cognitive deficits in various studies (Wu et al., 2015a; Cao et al., 2018; Fan et al., 2018). The NLRP3 inflammasome has been implicated as a crucial pro-inflammatory signaling mediator involved in mechanisms underlying inflammation. Assembly of the NLRP3 inflammasome triggers cleavage of caspase-1 and induces maturation of the proinflammatory cytokines IL-1β and IL-18, and subsequent cleavage of GSDMD and pyroptotic cell death (Man et al., 2017; Shi et al., 2017; Voet et al., 2019). Our results showed that surgery-induced upregulation of NLRP3 and cleaved caspase-1, and secretion of IL-1β and IL-18, in the hippocampus in aged mice, in agreement with the results of previous studies (Peng et al., 2019; Fu et al., 2020). Furthermore, surgery-induced upregulation of the GSDMD N domain, the key biomarker of pyroptosis, and the surgery group exhibited more ruptured and TUNEL-positive neurons, a morphological characteristic of pyroptosis. Collectively, our results provide important evidence that pyroptosis, as a result of NLRP3 inflammasome activation, is involved in the mechanism of PND.

Activation of the NLRP3 inflammasome and pyroptosis has been reported in various disorders, including inflammatory and neurodegenerative diseases (Man et al., 2017; Voet et al., 2019). However, the details on the mechanism of NLRP3 inflammasome activation by a variety of stimulators are largely unknown. Emerging evidence suggests that mitochondria play a vital role in NLRP3 inflammasome activation (Liu et al., 2018; Wei et al., 2019; Bock and Tait, 2020). Mitochondria are pivotal upstream targets that trigger NLRP3 inflammasome activation by providing intracellular stimulators, such as mitochondrial DNA, ROS, Ca^2+^, and cardiolipin (Heid et al., 2013; Iyer et al., 2013; Zhong et al., 2018). Also, mitochondria serve as a platform for inflammasome assembly, a process regulated by fission and fusion (Sandhir et al., 2017; Liu et al., 2018; Rovira-Llopis et al., 2018; Wei et al., 2019; Bock and Tait, 2020). Downregulation of mitofusin proteins or upregulation of DRP1 in mitochondria has been shown to lead to mitochondrial fragmentation and NLRP3 inflammasome activation (Li A. et al., 2016; Zhou et al., 2017). In agreement with these findings, our study demonstrated that surgery caused: (1) mitochondrial dysfunction, accompanied by NLRP3 inflammasome activation and pyroptosis, as evidenced by decreased ATP production and MMP levels, and increased ROS generation; and (2) abnormal mitochondrial morphology, as evidenced by an increased number of abnormal mitochondria and upregulation of mitochondrial DRP1. Therefore, our study suggested a correlation between mitochondrial dysfunction and NLRP3 inflammasome activation in PND.

Many chemicals targeting the mitochondria, such as mitochondrial ROS scavengers and mitochondrial division inhibitors, have been studied for their effects on inhibiting activation of the NLRP3 inflammasome (Dashdorj et al., 2013; Li Y. et al., 2016). Elamipretide is a mitochondrial-targeted peptide with multiple pharmacological properties that have been reported to improve mitochondrial function and decrease mitochondrial morphological damage in many neurological diseases (Wu et al., 2015a,b; Yin et al., 2016; Zhao et al., 2019). Here, we showed that elamipretide attenuated surgery-induced mitochondrial dysfunction by maintaining ATP production and MMP levels and decreasing ROS generation. Moreover, elamipretide protected the mitochondrial morphology against excessive fragmentation. Through protecting the mitochondria, elamipretide further attenuated surgery-induced NLRP3 inflammasome-caspase-1-dependent pyroptosis, neuroinflammation, neuronal damage, and synaptic dysfunction. Thus, elamipretide ameliorated surgery-induced cognitive deficits in aged mice. Our study provides a promising strategy for the treatment of cognitive deficits in PND involving mitochondrial dysfunction and pyroptosis.

Our findings have important clinical implications. Because of its superpower properties and safety, elamipretide has been investigated as a treatment for renal, cardiac, and skeletal muscle diseases in many clinical trials (Daubert et al., 2017; Saad et al., 2017; Karaa et al., 2018). Mitochondria are the initial and most vulnerable targets of anesthetics, as evidenced in both clinical and preclinical studies (Delogu et al., 2001; Muravchick and Levy, 2006), and mitochondrial dysfunction has been associated with the earliest stages of PND pathogenesis (Wu et al., 2015a; Chen et al., 2018; Zhao et al., 2019). Importantly, alleviation of mitochondrial dysfunction has been shown to relieve neuronal damage and ameliorate PND in patients (Chen et al., 2018), thus supporting the promise of therapeutics that target mitochondria in the treatment of PND.

This study has several limitations. First, we observed relatively short-term cognitive performance on the postoperative day 7. We did not investigate the long-lasting protective effect of elamipretide, although this will be an objective for future studies. Second, additional tests, such as Morris water maze tests, may be necessary to fully evaluate the hippocampus-dependent memory.

## Conclusion

In summary, the present study demonstrates that elamipretide treatment ameliorates neurocognitive deficits in PND mice. The protective effects of elamipretide are associated with mitochondria-protective properties, attenuation of neuroinflammation and neuronal pyroptosis, and improved synaptic integrity. Our research reveals novel insights into the mechanisms of PND and may provide a promising strategy for the treatment of PND.

## Data Availability Statement

All datasets presented in this study are included in the article.

## Ethics Statement

The animal study was reviewed and approved by the Animal Ethics Committee of Anhui Medical University.

## Author Contributions

EG and JW conceived and designed the experiments. YZ and LY performed experiments and data acquisition. YZ, LY, XC, JL, HW, and XL analyzed and interpreted the data. YZ and JW wrote the manuscript. All authors contributed to the article and approved the submitted version.

## Conflict of Interest

The authors declare that the research was conducted in the absence of any commercial or financial relationships that could be construed as a potential conflict of interest.
